# Genome-Wide Identification of *TCP* Family Transcription Factors in *Medicago truncatula* Reveals Significant Roles of *miR319*-Targeted *TCPs* in Nodule Development

**DOI:** 10.3389/fpls.2018.00774

**Published:** 2018-06-11

**Authors:** Hongfeng Wang, Hongwei Wang, Rong Liu, Yiteng Xu, Zhichao Lu, Chuanen Zhou

**Affiliations:** ^1^Key Laboratory of Plant Cell Engineering and Germplasm Innovation, Ministry of Education, School of Life Sciences, Shandong University, Qingdao, China; ^2^State Key Laboratory of Microbial Technology, Shandong University, Qingdao, China

**Keywords:** *Medicago truncatula*, *TCP* genes, *miR319*, expression analysis, nodule development

## Abstract

TCP proteins, the plant-specific transcription factors, are involved in the regulation of multiple aspects of plant development among different species, such as leaf development, branching, and flower symmetry. However, thus far, the roles of TCPs in legume, especially in nodulation are still not clear. In this study, a genome-wide analysis of *TCP* genes was carried out to discover their evolution and function in *Medicago truncatula*. In total, 21 *MtTCPs* were identified and classified into class I and class II, and the class II *MtTCPs* were further divided into two subclasses, CIN and CYC/TB1. The expression profiles of *MtTCPs* are dramatically different. The universal expression of class I *MtTCPs* was detected in all organs. However, the *MtTCPs* in CIN subclass were highly expressed in leaf and most of the members in CYC/TB1 subclass were highly expressed in flower. Such organ-specific expression patterns of *MtTCPs* suggest their different roles in plant development. In addition, most *MtTCPs* were down-regulated during the nodule development, except for the putative *MtmiR319* targets, *MtTCP3, MtTCP4*, and *MtTCP10A*. Overexpression of *MtmiR319A* significantly reduced the expression level of *MtTCP3/4/10A/10B* and resulted in the decreased nodule number, indicating the important roles of *MtmiR319*-targeted *MtTCPs* in nodulation. Taken together, this study systematically analyzes the *MtTCP* gene family at a genome-wide level and their possible functions in nodulation, which lay the basis for further explorations of *MtmiR319/MtTCPs* module in association with nodule development in *M. truncatula*.

## Introduction

TCP proteins, a small family of plant-specific transcription factors, was first described in 1999 and named after its initial members TEOSINTE BRANCHED1 (TB1) in maize (*Zea mays*), CYCLOIDEA (CYC) in snapdragon (*Antirrhinum majus*), and PROLIFERATING CELL FACTORS 1 and 2 (PCF1 and PCF2) in rice (*Oryza sativa*) (Luo et al., 1996; Doebley et al., 1997; Kosugi and Ohashi, 1997; Cubas et al., 1999). All TCP proteins contain a highly conserved TCP domain which is a 59-amino-acid non-canonical basic-helix-loop-helix (bHLH) motif at the N-terminus and functions in DNA binding, protein–protein interaction, and protein nuclear localization (Kosugi and Ohashi, 2002; Aggarwal et al., 2010; Martin-Trillo and Cubas, 2010; Dhaka et al., 2017). So far, *TCP* genes have been identified in various plant species including *Arabidopsis*, rice, tomato, tobacco, and strawberry as shown in Supplementary Figure S1 (Palatnik et al., 2003; Li et al., 2005; Aguilar-Martinez et al., 2007; Ori et al., 2007; Yao et al., 2007; Schommer et al., 2008; Nag et al., 2009; Giraud et al., 2010; Koyama et al., 2010b; Sarvepalli and Nath, 2011; Yang et al., 2012; Ma et al., 2014; Parapunova et al., 2014; Wang et al., 2015b; Chen et al., 2016; Lin et al., 2016; Ma et al., 2016; Wei et al., 2016; Zhou et al., 2016; Du et al., 2017; Xie et al., 2017).

The TCP family can be classified into two classes: class I (also known as PCF class or TCP-P class) and class II (also known as TCP-C class) according to the amino acid sequences of the TCP domain (Kosugi and Ohashi, 2002; Navaud et al., 2007; Martin-Trillo and Cubas, 2010). The class II can be further divided into two unlike subclasses: CIN and CYC/TB1 (Crawford et al., 2004; Howarth and Donoghue, 2006; Martin-Trillo and Cubas, 2010). The difference between the class I and class II TCP proteins is a four-amino-acid deletion in the TCP domain in class I. Besides the TCP domain, all members of the CYC/TB1 TCP proteins contain an arginine-rich R domain with unknown function, which is speculated to facilitate protein–protein interaction (Cubas et al., 1999).

Class I TCPs mainly participate in promoting cell proliferation and plant growth, such as the *PCF1*/*PCF2* in rice and *TCP20* in *Arabidopsis* (Kosugi and Ohashi, 1997; Li et al., 2005). Most loss-of-function mutants of class I *TCP* genes do not exhibit obvious phenotypic defects due to the genetic redundancy. In *Arabidopsis*, class I genes *TCP14* and *TCP15* have redundant functions in the regulation of internode length, leaf shape, seed germination, and endoreduplication (Kieffer et al., 2011; Uberti-Manassero et al., 2012; Resentini et al., 2015). *TCP20* appears to participate in different developmental processes, such as jasmonic acid (JA) biosynthesis and leaf senescence (Li et al., 2005; Danisman et al., 2012, 2013). Meanwhile, *TCP9* and *TCP19* act redundantly with *TCP20* and positively regulate leaf senescence through the JA signaling pathway (Danisman et al., 2012, 2013). *TCP16* mainly expresses in developing microspores and plays a crucial role at the early stage of pollen development (Takeda et al., 2006). Moreover, *TCP7, TCP8, TCP22*, and *TCP23* show similar expression patterns in young leaves and have genetic redundancy in regulation of leaf development (Aguilar-Martinez and Sinha, 2013).

MicroRNAs (MiRNAs) are small non-coding RNAs (20–24 nucleotides in length) that can complementarily bind to their target mRNAs and reduce their expression levels (Reinhart et al., 2002; Voinnet, 2009; Rogers and Chen, 2013). Recent evidences indicate that miRNAs are essential genetic regulators and play vital roles in plant development (Chen, 2008). In the CIN subclass, five *Arabidopsis TCP* genes *TCP2, TCP3, TCP4, TCP10*, and *TCP24* are the targets of *miR319* and have been involved in regulation of cell division to control leaf morphogenesis (Palatnik et al., 2003; Bresso et al., 2017). In tomato, high levels of *miR319* or low *LA* activity cause an excess of cell expansion, resulting in the super compound leaves (Ori et al., 2007). Meanwhile, the CYC/TB1 subclass *TCPs* involve in floral dorsoventral asymmetry development and shoot branching, such as *CYC* in *Lotus japonicus*, snapdragon and *Pisum sativum* (Luo et al., 1996, 1999; Almeida and Galego, 2005; Feng et al., 2006; Wang et al., 2008, 2010; Li et al., 2010; Weng et al., 2011; Xu et al., 2013) and *TB1* in maize (Doebley et al., 1995). In *Arabidopsis*, two orthologs of maize *TB1, BRANCHED1* (*BRC1, TCP18*) and *BRANCHED2* (*BRC2, TCP12*), are expressed in axillary buds. Both of them are involved in suppressing axillary bud outgrowth and loss-of-function in these genes results in the increased shoot branching (Aguilar-Martinez et al., 2007; Finlayson, 2007). *TCP1*, a member of the CYC/TB1 subclade, is implicated in the control of brassinosteroid (BR) biosynthesis and the regulation of longitudinal elongation in plant (Guo et al., 2010; Koyama et al., 2010b).

*Medicago truncatula* is a model species for legume genetics and functional genomics study. However, the *TCP* family in *M. truncatula* has not been characterized, and their roles are still unknown, especially in nodulation. In this study, a global analysis of the *TCP* gene family in *M. truncatula* was carried out. Twenty-one *MtTCP* genes were identified, and their phylogenetic relationship, gene structure, protein motifs, chromosomes locations, and transcriptional levels in different organs were analyzed. Furthermore, we found that the expression patterns of *MtTCP* genes are different during the nodule development. Based on the time course of *MtTCPs* expression in nodules, *MtTCP3, MtTCP4*, and *MtTCP10A*, three of four target genes of *MtmiR319*, were significantly up-regulated after inoculation with rhizobia. Overexpression of *MtmiR319A* led to the decreased nodule number, which further confirms the significant roles of *MtmiR319-*targeted *MtTCPs* in nodulation. Therefore, this study provides detailed information of the *MtTCPs* classification and throws some light into the function of *MtTCPs* involved in nodule development in *M. truncatula*.

## Materials and Methods

### Plant Material and Root Nodule Induction

For nodule induction, wild-type (ecotype R108) and *35S:MtmiR319A* seeds were transferred to plastic seedling holes containing a 3:1 ratio of pearlite/sand under a 16-h/8-h light/dark at 22°C and 60% relative humidity. The *S. meliloti* 1021 strain harboring the *lacZ* reporter gene (Boivin et al., 1990) was cultured in TY medium supplemented with 6 mmol l^-1^ calcium chloride, 200 μg ml^-1^ streptomycin, and 10 μg ml^-1^ tetracycline. The *S. meliloti* culture was shaken at 220 rpm and 28°C overnight. The *S. meliloti* growth was monitored by measuring optical density at a wavelength of 600 nm (OD_600_) until the OD_600_ value reached to 1.0. Five-day-old seedling was inoculated with 5 ml of *S. meliloti* 1021 strain suspension with OD_600_ of 0.1 (adjusted OD_600_ value from 1.0 to 0.1 by dilution). At 3 weeks post-inoculation, seedling roots, nodule number, and the fresh weight of shoot/root/nodule were analyzed. For nodulation time course assays, nodules in wild-type were harvested at 7, 14, and 21 days post inoculation (dpi). For LacZ staining, LacZ activity of nodules was performed as previously described (Boivin et al., 1990).

### Identification and Phylogenetic Analysis of the *TCP* Genes in *M. truncatula*

To identify all *TCP* genes in *M. truncatula*, we use the 24 known *TCP* genes from *Arabidopsis* and 21 *TCP* genes from rice to perform protein to protein BLAST in *Medicago truncatula* resource website^1^ (Martin-Trillo and Cubas, 2010; Shi et al., 2016). *TCP* genes in *Arabidopsis* were downloaded from TAIR^2^ online. *TCP* genes in rice were downloaded from the Rice Genome Annotation Project^3^ online. Based on the searched TCP chromosome locus, we downloaded the genome sequence from Medicago truncatula Genome Database^4^. The BLASTP parameters are *E*-value = 10, top hits number = 30. In total, 27 MtTCPs sequences with annotations were obtained. As each of *Medtr2g090960, Medtr1g038650*, and *Medtr2g006150* has two splice variants, and *Medtr1g101810* has four splice variants. We selected splice variant 1 for study, and other splice variants were excluded. Therefore, 21 independent *MtTCP* genes were identified in *M. truncatula*. Then, the *MtTCP* sequences were confirmed in the Plant Transcription Factor Database (PlantTFDB^5^) online. To study the phylogenetic relationships among *TCP* genes in *M. truncatula, Arabidopsis*, and rice, 21 identified MtTCP proteins in *M. truncatula* and 24 *Arabidopsis* AtTCP proteins and 21 rice OsTCP proteins were used to generate the phylogenetic tree. Multiple sequence alignments were executed using CLUSTALW^6^ online. Then, the phylogenetic trees were constructed using MEGA7.1 by the Neighbor Joining (NJ) method with 1000 bootstrap replicates in p-distance model. Since the TCP family in *Arabidopsis* has been identified and studied well, we named *MtTCPs* based on the phylogenetic relationship with *AtTCPs* and Blast analysis against the *Arabidopsis* genome in TAIR.

### Gene Structure Analysis of *MtTCP* Genes

To analyze the gene structures and exon–intron organization of the *MtTCP* genes, we downloaded the *MtTCP* genes genomic sequences and structural information from the Medicago truncatula Genome Database. Then, the *MtTCP* genomic sequences and CDS sequences were aligned using the gene structure display server 2.0 (GSDS) online^7^ to generate the diagrams of exon–intron structures.

### Conserved Domains and Motif Analysis in MtTCP Proteins

Conserved motifs in MtTCP proteins were analyzed with the online Multiple Em for Motif Elicitation (MEME) program^8^. The optimized MEME parameters were as follows: repetition number, any; maximum motif width, 200; minimum motif width, 6; and maximum motif number, 20. Multiple protein sequences alignment was carried out with Jalview software^9^.

### RNA Isolation, Gene Expression Analysis, and Statistical Analysis

To detect the expression patterns of *TCP* genes in *M. truncatula*, total RNA was extracted from the young leaves (folded leaves developed on the first and second internodes), mature leaves (fully expanded leaves developed on the older internodes), roots, stems, flowers, shoot buds, pods, and nodules at the different stages using the Trizol-RT Reagent (Molecular Research Center, INC), according to the manufacturer’s instructions. RNA quantitative and qualitative measurements were achieved using Nanodrop 2000 Spectrophotometer (NanoDrop Technologies, United States). Three micrograms of total RNA from each organs/tissue was reverse-transcribed into cDNA using the Roche RNA Reverse Transcription Kit (Roche, United States). The qRT-PCR was performed on Bio-Rad CFX Connect^TM^ using Roche SYBR-green fluorescence dye (FastStart Essential DNA Green Master). The relative expression levels were calculated using 2^-ΔΔCT^ method. For all qRT-PCR analysis, triplicate biological samples were collected. The *MtUBI* gene was selected as internal control for normalization. *T*-test was used to estimate if the difference is significant in analysis of gene expression level, fresh weight, and nodule number.

### Subcellular Localization of *MtTCPs* and Cloning of the *MtmiR319A* Gene

For subcellular localization, PCR was performed to amplify the coding sequences of *MtTCP3, MtTCP4, MtTCP10A*, and *MtTCP10B* genes using gene-specific primers which are listed in Supplementary Table S1. Then, the PCR products were purified and cloned into pEarleyGate 103 (Earley et al., 2006) using the Gateway LR reaction (Invitrogen). The *Agrobacterium tumefaciens* EHA105 strain harboring relevant plasmids was transformed into tobacco epidermal cells. The MtTCP-GFP fusion proteins were examined using a confocal laser scanning microscope LSM 700 (Zeiss). To construct the *MtmiR319A* overexpression vector, *MtmiR319A* genomic sequence was PCR amplified and transferred into pEarleyGate 100. The leaves of the wild-type were transformed with the EHA105 strain harboring the *MtmiR319A* overexpression vector.

### Identification of the *MtmiR319* and Prediction of the *MtmiR319* Target Genes

The mature *MtmiR319* sequences were obtained on miRBase Database^10^ online. To predict *MtmiR319* target sites, full length of *MtTCPs* coding sequences was analyzed using the psRNATarget (A Plant Small RNA Target Analysis^11^) online. The mature *miR319* sequences of rice were obtained on miRBase Database. The mature *miR319* sequences of *Arabidopsis* were downloaded on TAIR^12^ online and confirmed on miRBase Database.

## Results

### Identification of *TCP* Genes in *M. truncatula*

To identify TCP proteins in *M. truncatula*, the TCP protein sequences of *Arabidopsis* and rice were used to BLAST search against the Medicago truncatula Genome Database^13^ and Plant Transcription Factor Database (PlantTFDB^14^). A total of 21 putative MtTCP sequences were obtained, which all contained the conserved TCP domain. The length of the protein sequences of 21 MtTCPs ranged from 205 to 489 amino acids. The gene locus, exon number, amino acid length, molecular weight (Mw), isoelectric point (pI), type, and chromosome location of *MtTCPs* are listed in **Table 1**. Based on the gene locus data, these *MtTCPs* were unevenly located on different chromosomes. Five *MtTCPs* were located on Chr1. Four *MtTCPs* were located on Chr4. Furthermore, the Chr2 and Chr7 chromosomes each had three *MtTCPs*. The other *MtTCPs* were distributed on Chr3, Chr5, Chr6, and Chr8, respectively (**Table 1**).

**Table 1:** TCP gene family in *M. truncatula.*

Name	Locus	CDS	Extrons	Length (aa)	MW (kDa)	pI	Type	Chromosomes location
*MtTCP1A*	Medtr6g017055	1230	2	409	46,096.18	9.436	CYC/TB1	chr6:6710788..6713192 (+)
*MtTCP1B*	Medtr7g018500	1125	2	374	42,704.88	6.966	CYC/TB1	chr7:6031315..6033168 (+)
*MtTCP3*	Medtr2g078200	885	1	294	32,176.38	7.084	CIN	chr2:32505911..32507609 (+)
*MtTCP4*	Medtr8g463380	1302	1	433	47,448.71	6.953	CIN	chr8:22326723..22329800 (+)
*MtTCP5A*	Medtr3g026050	1158	1	385	43,448.1	7.716	CIN	chr3:7969037..7970349 (+)
*MtTCP5B*	Medtr4g109660	921	1	306	33,901.83	9.198	CIN	chr4:45588798..45589718 (+)
*MtTCP7*	Medtr1g038650	708	1	235	25,831.56	8.251	PCF	chr1:14248871..14250840 (-)
*MtTCP9*	Medtr8g033070	1002	1	333	36,244.28	9.491	PCF	chr8:12681955..12683583 (+)
*MtTCP10A*	Medtr2g090960	990	1	329	36,325.62	6.322	CIN	chr2:39075847..39078457 (-)
*MtTCP10B*	Medtr4g079580	1008	1	335	37,656.05	6.327	CIN	chr4:30767465..30768533 (-)
*MtTCP11*	Medtr1g063870	618	1	205	21,958.6	7.447	PCF	chr1:28041642..28042798 (+)
*MtTCP12*	Medtr1g103380	1470	2	489	56,235	7.966	CYC/TB1	chr1:46794627..46797920 (-)
*MtTCP13*	Medtr7g015010	1056	1	351	39,686.79	8.564	CIN	chr7:4515349..4517203 (+)
*MtTCP14*	Medtr5g039600	1251	1	416	44,227.9	6.726	PCF	chr5:17427951..17429938 (-)
*MtTCP15*	Medtr4g108370	1314	1	437	47,447.01	7.818	PCF	chr4:44959266..44961490 (-)
*MtTCP17*	Medtr6g015350	1002	1	333	37,706.17	6.671	CIN	chr6:5010976..5013085 (+)
*MtTCP18*	Medtr4g111935	1167	2	388	44,497.4	8.813	CYC/TB1	chr4:46279251..46280786 (-)
*MtTCP19*	Medtr1g101810	1104	1	367	38,729.49	5.251	PCF	chr1:45965265..45966764 (+)
*MtTCP20*	Medtr7g028160	855	1	284	30,714.79	8.664	PCF	chr7:9483450..9485316 (-)
*MtTCP21*	Medtr1g114380	774	1	257	27,430.59	9.492	PCF	chr1:51614461..51616519 (+)
*MtTCP22*	Medtr2g006150	1464	1	487	51,820.53	6.581	PCF	chr2:468724..471446 (-)

### Phylogenetic Analysis and Classification of *TCP* Genes in *M. truncatula*

**FIGURE 1:**
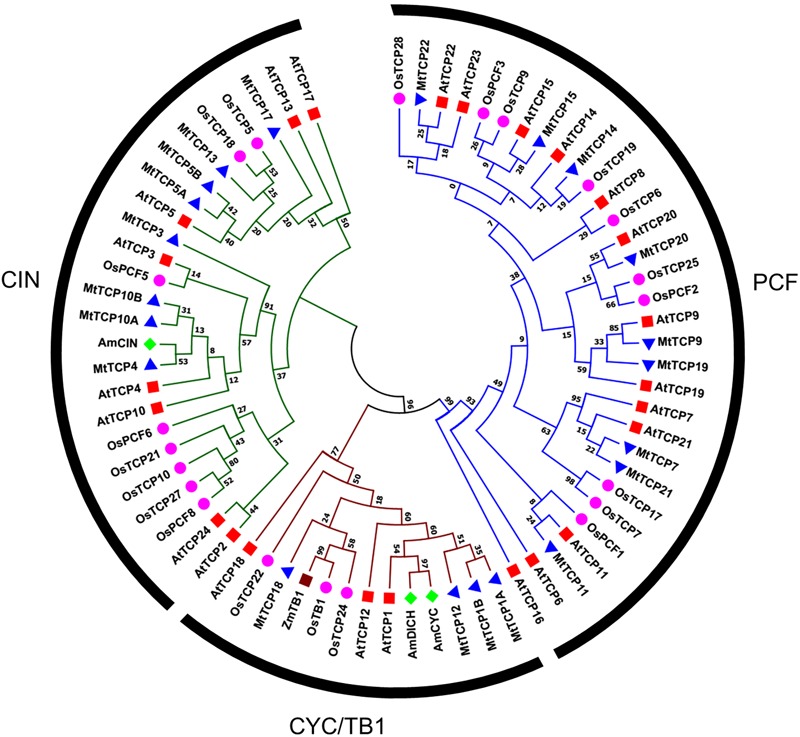
Phylogenetic relationships of TCP transcription factors from *M. truncatula* (MtTCP), *Arabidopsis* (AtTCP), rice (OsTCP), maize (ZmTB1), and *A. majus* (AmCIN/AmCYC/AmDICH). The phylogenetic tree was constructed using 70 full-length protein sequences from *M. truncatula* (21), *Arabidopsis* (24), rice (21), maize (1), and *A. majus* (3) by the Neighbor-Joining method in MEGA 7.1 with 1000 bootstrap replicates. The branched lines of the subtrees are colored to indicate different TCP subclasses.

**FIGURE 2:**
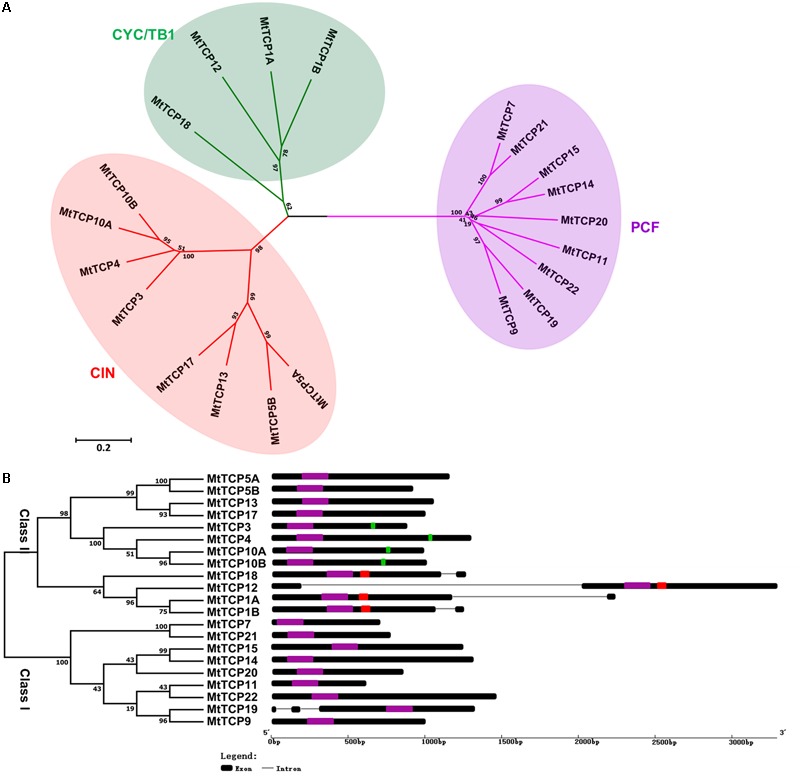
Phylogenetic tree and exon/intron structural analysis of all TCP transcription factors in *M. truncatula*. **(A)** Phylogenetic tree was generated based on the full-length protein sequences of all *MtTCP* genes. Multiple protein sequences were aligned using Clustal W and the phylogenetic trees were constructed using MEGA7.1 by the Neighbor-Joining method and the bootstrap test was performed with 1000 replicates. **(B)** The exon/intron organization of *MtTCP* genes in *M. truncatula*. Exons and introns of *MtTCP* genes were indicated by black rounded rectangles and black lines, respectively. The TCP domain, R domain, and *MtmiR319* recognition sequence are indicated in purple, red, and green rounded rectangles, respectively. The scale was referred to the lengths of the genes.

To evaluate the evolutionary and phylogenetic relationships among the TCP transcription factor families among species, a total of 70 TCP protein sequences, including 21 MtTCPs, 24 AtTCPs, and 21 OsTCPs, 1 ZmTCP (maize, TB1), and 3 AmTCPs (*A. majus*, AmCIN/AmCYC/AmDICH) were collected to construct an unrooted phylogenetic tree using MEGA7.1 by the NJ method with 1000 bootstrap replicates. According to its classification in *Arabidopsis*, the MtTCPs could also be classified into two TCP classes. Class I contained nine members of MtTCPs. The rest of MtTCPs were grouped as Class II which can be further divided into two subclasses: the CIN (eight members) and the CYC/TB1 (four members) (**Figures 1, 2A**). Then, the TCP domains of each MtTCP were identified and used for further phylogenetic analysis (Supplementary Figure S2). The phylogenetic trees derived from full-length protein sequences and TCP domains are essentially the same, indicating the evolutionary conservation among these TCPs.

### Gene Structure, Conserved Motifs, and Recognition Sequence of *miR319*

To get the understanding of the diversification of *MtTCPs*, the structure and number of exon/intron of *MtTCPs* were analyzed (**Figure 2B**). We investigated the exon/intron organization of individual *MtTCPs* by aligning the CDS sequences and corresponding genomic DNA sequences. The class I *MtTCPs* displayed a conserved exon–intron organization: eight of nine *MtTCPs* had no intron, only *MtTCP19* possessed two introns. However, the class II *MtTCPs* showed different number of introns. All the CYC/TB1 subclass *MtTCPs* contained one more intron than those of CIN subclass genes (**Figure 2B**).

**FIGURE 3:**
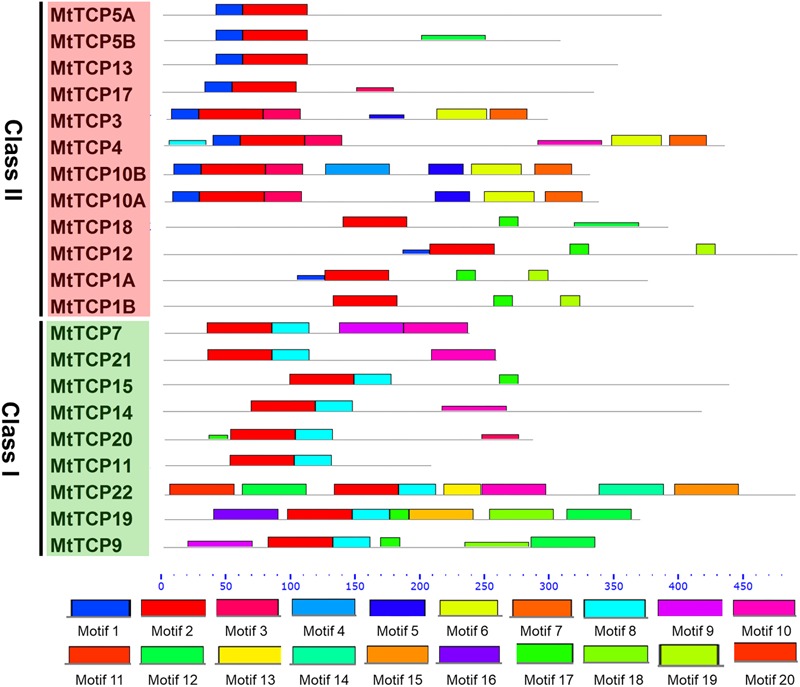
Motif composition of MtTCP proteins. The motifs in Medicago TCP proteins were indicated using MEME online tool. The motifs in the MtTCP proteins are denoted by rectangles with different colored boxes. The conserved TCP domains were shown by red boxes. The positions and lengths of motifs in the MtTCP proteins were identified by the colored boxes.

**FIGURE 4:**
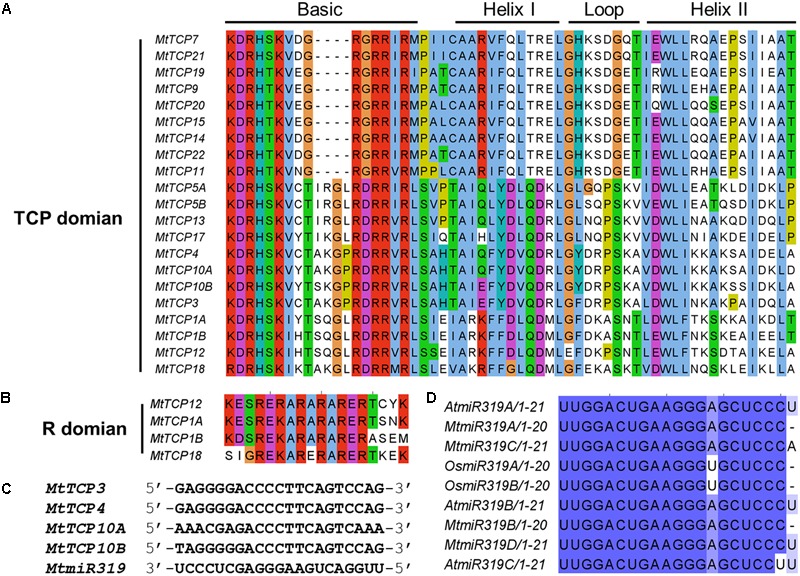
Multiple sequence alignment of MtTCP transcription factors and the *MtmiR319*-targeted *MtTCPs*. **(A)** Alignment of TCP domain of the 21 TCP proteins in *M. truncatula*. Amino acids that are conserved throughout are shaded in different colors. Conserved domains, including Basic, Helix I, Loop, and Helix II, are shown at the top. **(B)** Alignment of R-domain of the CYC/TB1 subclass members. Conserved amino acids are shaded in different colors. Multiple sequence alignment was carried out using ClustalW and visualized with Jalview software. **(C)** Alignment of putative target regions for *MtmiR319* binding in CIN subclass *MtTCP* genes. **(D)** Mature *miR319* sequences in rice, *Arabidopsis*, and *M. truncatula*. Shaded zones show the conserved sequences.

To obtain a better understanding of the evolutionary relationships of the TCP proteins in *M. truncatula*, the motifs of MtTCPs were analyzed (**Figure 3**). In total, 20 motifs were identified in MtTCPs using the online MEME tool. As expected, all the 21 MtTCPs displayed a highly conserved TCP domain. Analysis of the phylogenetic tree and the alignment of the TCP domains showed that MtTCP proteins can be divided into two classes (**Figures 2B, 4A** and Supplementary Figure S3), as for all species so far. The conserved R domain was only found in MtTCP1A, MtTCP1B, MtTCP12, and MtTCP18, which are the members of CYC/TB1 subclass in Class II (**Figures 2B, 4B** and Supplementary Figure S3).

In *Arabidopsis*, five members of the Class II *MtTCPs* subfamily (*AtTCP2, AtTCP3, AtTCP4, AtTCP10*, and *AtTCP24*) are post-transcriptionally regulated by *AtmiR319* (Palatnik et al., 2003). In *M. truncatula*, the evolutionarily closest homologs of these *Arabidopsis* genes are *MtTCP3, MtTCP4, MtTCP10A*, and *MtTCP10B*, which contained a putative recognition site of *MtmiR319* (**Figures 2B, 4C**). We searched the miRBase Database and found four *MtmiR319* (*MtmiR319A-D*) in *M. truncatula* genome (Supplementary Figure S4). The alignment of multiple *miR319* mature sequences showed that the *miR319-TCP* regulation module was highly conserved among species (**Figure 4D**). Although mismatches existed at 3′ of the *MtmiR319* and 5′ of the targeted *MtTCP* mRNA, core target sequences were conserved. These data suggest that *MtTCP3, MtTCP4, MtTCP10A*, and *MtTCP10B* probably play the roles in an *MtmiR319-*regulated manner in *M. truncatula*.

### Distinct Expression Profiles of *MtTCPs* in *M. truncatula*

**FIGURE 5:**
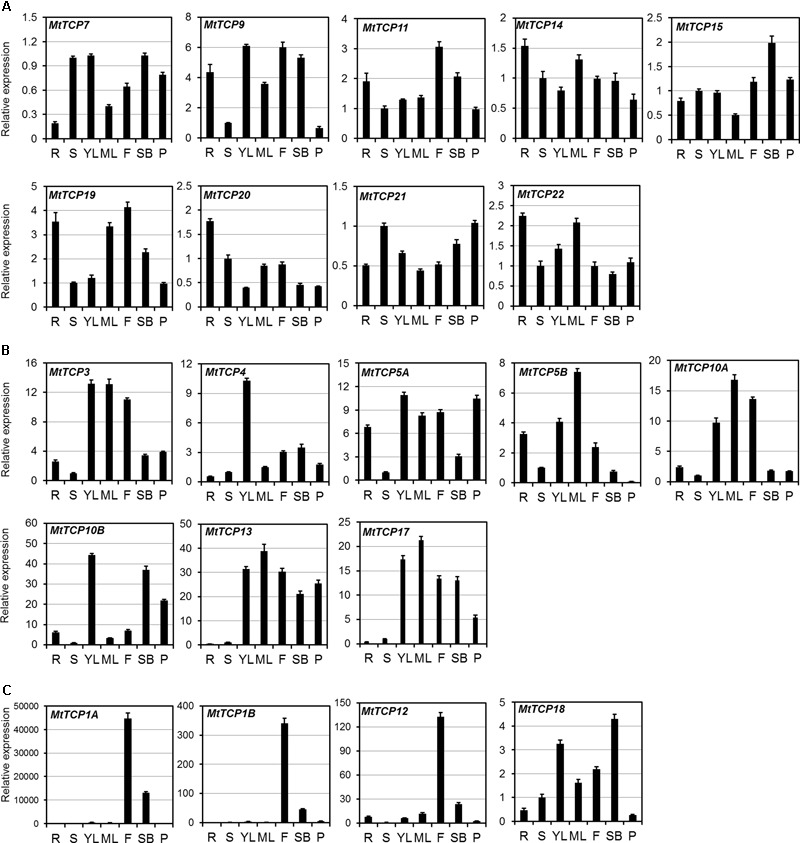
The expression patterns of 21 *MtTCP* genes in different organs. **(A)** The expression level of *MtTCPs* of Class I **(A)**, Class II CIN clade **(B)**, and CYC/TB1 clade **(C)** in roots (R), stems (S), young leaves (YL), mature leaves (ML), flowers (F), shoot buds (SB), and pods (P), were detected by qRT-PCR. The relative expression level was normalized using *MtUBI*. Values are the mean and SD of three replicates.

To investigate the tissue-specific expression profiles of *MtTCP* genes, their relative expression levels in different organs, including young leaves, mature leaves, roots, stems, flowers, shoot buds, and pods, were analyzed by quantitative real-time PCR (qRT-PCR). As indicated in **Figure 5**, some Class I *MtTCP* genes were differentially expressed in the different organs, while other Class I *MtTCP* genes showed similar expression patterns among different organs. For example, *MtTCP9, MtTCP11*, and *MtTCP19* were highly expressed in root and flower, whereas *MtTCP7, MtTCP20*, and *MtTCP21* were expressed at very low level in all organs examined. This finding implies that those Class I *MtTCP* genes probably play different roles during plant growth and development. Most CYC/TB1 subclass *MtTCPs* (*MtTCP1A, MtTCP1B*, and *MtTCP12*) showed relatively weak expression in the root, stem, leaf, and pod, but highly expressed in flower, implying their specific roles in flower development. While another CYC/TB1 subclass *MtTCP* gene (*MtTCP18*) was highly expressed in leaf, flower, and shoot bud, but lowly expressed in root, stem, and pod. In contrast, the expression levels of all the *MtmiR319* target CIN subclass *MtTCPs* (*MtTCP3, MtTCP4, MtTCP10A*, and *MtTCP10B*) were very high in young leaf, indicating that they may play an important role in leaf development. The non-*MtmiR319* target CIN *MtTCPs, MtTCP13*, and *MtTCP17* showed similar expression patterns. Both of them were strongly expressed in leaf, flower, shoot bud, and pod, but weakly expressed in root and stem, implying their similar roles in plant developmental processes. *MtTCP5A* and *MtTCP5B* were highly expressed in root, leaf, and flower, but weakly expressed in stem. The qRT-PCR data suggest that *MtTCPs* may be involved in different aspects of plant growth and development. However, further studies are still needed to uncover the functional divergence of *MtTCP* genes in *M. truncatula*.

### *MtmiR319*-Targeted *MtTCPs* Play the Vital Roles in Nodule Development

**FIGURE 6:**
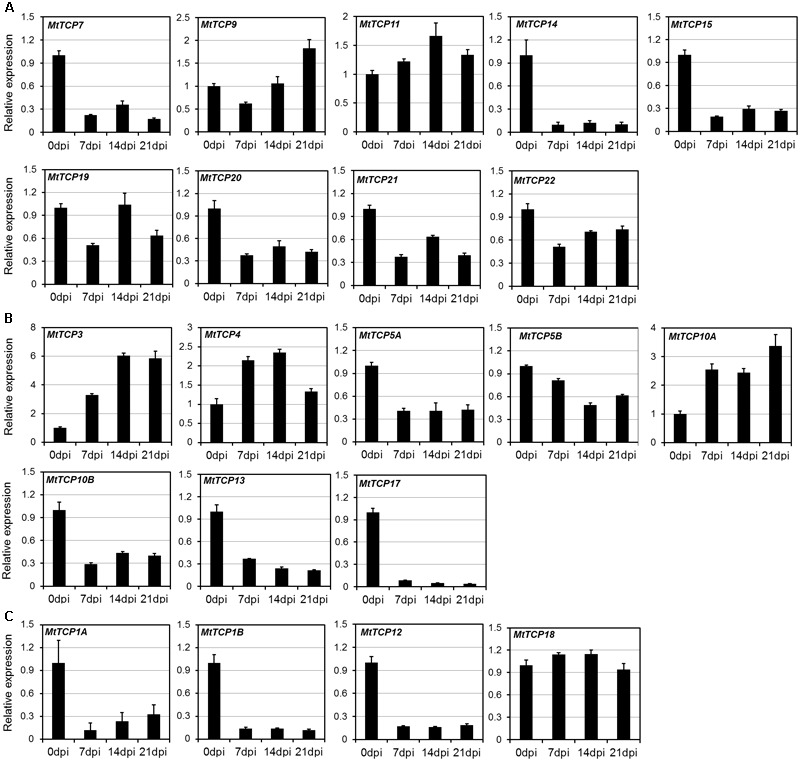
Expression pattern of *MtTCPs* in different developmental stages of nodule using qRT-PCR. The relative expression level of *MtTCPs* of Class I **(A)**, Class II CIN clade **(B)**, and CYC/TB1 clade **(C)** in 0, 7, 14, and 21 days post inoculation (dpi) were detected by qRT-PCR. The expression profiles were normalized using *MtUBI*. Values are the mean and SD of three replicates.

Roots play an important role in plant growth and development, such as nutrient and water uptake and the establishment of symbiotic interactions (Lopez-Bucio et al., 2003). It is important for legume species, since it can adapt to low nitrogen (N) by symbiosis with nitrogen-fixing rhizobia in nodules (Graham et al., 1981; Carroll et al., 1985; Caetanoanolles and Gresshoff, 1990). To elucidate the roles of *MtTCPs* in nodule development, the qRT-PCR was conducted to measure their relative expression levels at the different stages of nodule development (**Figure 6**). Nodules were harvested at 7, 14, and 21 dpi inoculated with *S. meliloti* 1021 strain, roots without inoculation as control (Supplementary Figure S5). The expression levels of most *MtTCPs* were down-regulated at different developmental stages of nodules, compared with those in root of 0 dpi. However, the expression levels of *MtmiR319*-targeted genes, *MtTCP3, MtTCP4*, and *MtTCP10A*, were significantly increased. This finding suggests that *MtmiR319-MtTCPs* module is probably involved in nodule development.

**FIGURE 7:**
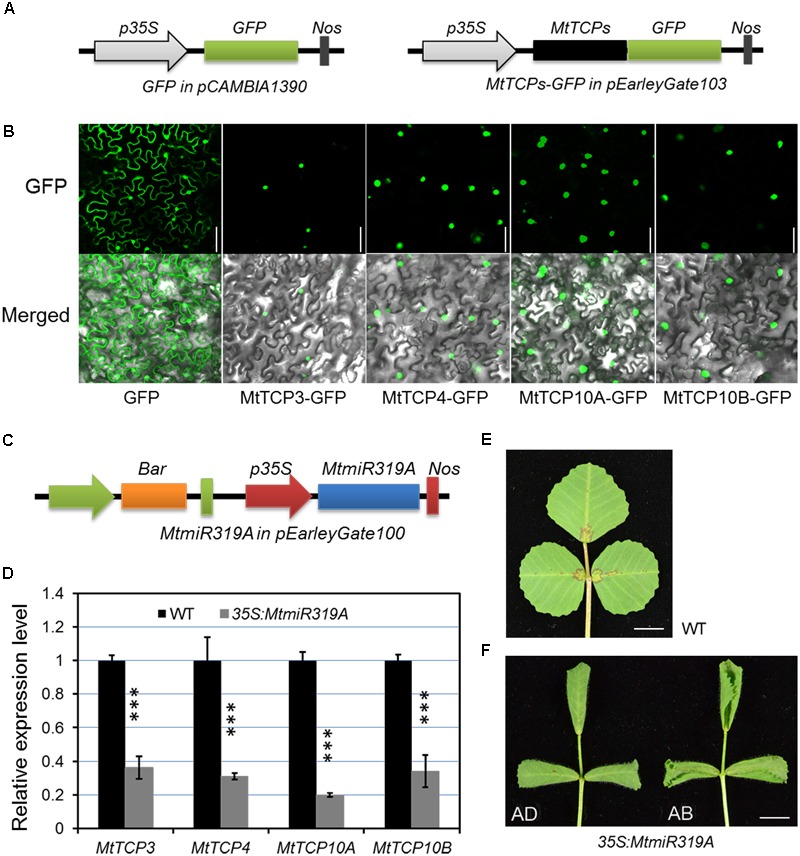
Subcellular localization of the four *MtmiR319-*targeted *MtTCP* genes and phenotypic effects of the *35S:MtmiR319A*. **(A)** Schematic illustration of vectors used in subcellular localization analysis. Black box represents the *MtTCP* genes. Green boxes represent green fluorescence protein (GFP). The *MtmiR319*-targeted *MtTCP* genes were cloned to pEarleyGate 103 vector, in which GFP was fused at the C-terminus. **(B)** The subcellular localization of four *MtmiR319*-target MtTCPs-GFP fusion proteins. Free GFP as control. Bar = 100 μm. **(C)** Schematic illustration of vectors used for *MtmiR319A* overexpression. **(D)** Relative expression level of the target genes in the T1 generation of *35S:MtmiR319A* plants. Transcript levels were measured by qRT-PCR. Values are the means and SD of three biological replicates. *T* = 2 and *^∗∗∗^P* < 0.001. **(E)** The leaf of wild-type. **(F)** The leaf of *35S:MtmiR319A* plant. AD, adaxial side of leaf; AB, abaxial side of leaf. Bars = 5 mm in **(E,F)**.

To further evaluate the possible function of *MtmiR319-MtTCPs* module, the subcellular localization of the *MtmiR319*-targeted *MtTCPs* was performed. MtTCP3, MtTCP4, MtTCP10A, and MtTCP10B were, respectively, fused with GFP, and transformed into tobacco epidermal cells. Based on the observation of green fluorescence signal, these *MtmiR319*-target *MtTCPs* were located in the cell nucleus, suggesting that they are functional transcription factors (**Figures 7A,B**). Then, the *MtmiR319A* sequence was introduced into wild-type plants under the regulation of the cauliflower mosaic virus 35S promoter and the stable transgenic plants were obtained. The expression levels of *MtTCP3, MtTCP4, MtTCP10A*, and *MtTCP10B* were significantly down-regulated in the *35S:MtmiR319A* plants, indicating that these four *MtTCPs* are the targets of *MtmiR319A* (**Figures 7C,D**). *35S:MtmiR319A* transgenic plant displayed downward-curled leaves with pronounced serrations on the leaf margin (**Figures 7E,F**), indicating that *MtmiR319*-target *MtTCPs* play the conserved roles in leaf development, similar to their functions in *Arabidopsis*.

**FIGURE 8:**
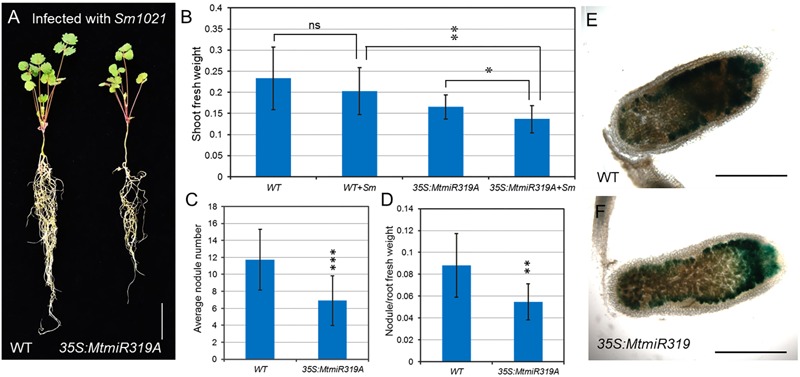
*MtmiR319*-targeted *MtTCP* genes are involved in symbiotic root nodule development. **(A)** Phenotype of wild-type and *35S:MtmiR319A* plants after 3 weeks inoculated with *S. meliloti* strains 1021. Bar = 1 cm. **(B)** The shoot fresh weight of 3-week-old wild-type and *35S:MtmiR319A* plants with inoculation (+Sm) or without inoculation. **(C)** The average of nodule number in wild-type and *35S:MtmiR319A* plants. **(D)** The ratio of nodule/root fresh weight in wild-type and *35S:MtmiR319A* plants. **(E,F)** The 21 dpi nodules in wild-type and *35S:MtmiR319A* plants were sliced into sections for β-galactosidase staining. Bar = 2 mm in **(E,F)**. Values are the means ± SD (*n* = 20). *T* = 2, *^∗^P* < 0.05, *^∗∗^P* < 0.01, *^∗∗∗^P* < 0.001.

To better characterize their roles in nodule development, wild-type and *MtmiR319A*-overexpressing plants were inoculated with *S. meliloti* 1021 strain. After 3 weeks of inoculation, the *MtmiR319A*-overexpressing plants exhibited reduced shoot fresh weight, nodule numbers, and decreased ratio of nodule/root fresh weight compared with those in wild-type (**Figures 8A–D** and Supplementary Figure S6). However, there was no obvious difference in nodule size and shape between wild-type and *MtmiR319A*-overexpressing plants (Supplementary Figure S7). After 3 weeks of inoculation, the nodules were harvested for histological studies. The longitudinal sections of nodules were stained for β-galactosidase activity to visualize the bacteria, since the *S. meliloti* 1021 strain carried a constitutively expressed hemA/lacZ fusion. The staining in the distal zone of wild-type nodules was much darker than that of *MtmiR319A*-overexpressing nodules, implying less functional zone of nitrogen fixation exists in *MtmiR319A*-overexpressing nodules (**Figures 8E,F**). These results indicate that *MtmiR319/MtTCPs* module is essential not only for nodule number, but also for nodule symbiosis.

## Discussion

TCP family transcription factors play important roles in multiple processes during plant growth and development, such as leaf development, flower development, phytohormone biosynthesis, endoreduplication, circadian clock, and shoot lateral branching (Palatnik et al., 2003; Takeda et al., 2003; Aguilar-Martinez et al., 2007; Damerval et al., 2007; Ori et al., 2007; Nag et al., 2009; Guo et al., 2010; Koyama et al., 2010a; Wang et al., 2010, 2015a,b; Sarvepalli and Nath, 2011; Shleizer-Burko et al., 2011; Danisman et al., 2012; Li et al., 2012; Aguilar-Martinez and Sinha, 2013; Li and Zachgo, 2013; Niwa et al., 2013; Tao et al., 2013; Das Gupta et al., 2014; Schommer et al., 2014; Lopez et al., 2015; Lucero et al., 2015; Yang et al., 2015; Alvarez et al., 2016; Danisman, 2016; Nicolas and Cubas, 2016; Uberti-Manassero et al., 2016; Wei et al., 2016; Bresso et al., 2017; Koyama et al., 2017). In this study, 21 *MtTCPs* were identified in *M. truncatula*, which were distributed on eight chromosomes with different densities.

Generally, genes within same class/clade shared the similar gene structure. Through exon–intron organization analysis of *MtTCP* family members, except for *MtTCP19*, the class I and CIN clade *TCP* genes show similar exon–intron structure that possessed only a single exon. While all members in CYC/TB1 clade display similar exon–intron organization with one exon and one intron. Such exon–intron structure similarity/variation might be caused by single intron loss or gain during the process of gene structure evolution. Protein sequence motif analysis revealed that MtTCPs within the same class/clade exhibit similar motif distribution. Moreover, TCP domains are highly conserved in all members of the MtTCP family, suggesting that the MtTCPs may share similar DNA binding capacity (Kosugi and Ohashi, 1997; Cubas et al., 1999).

It is noted that all class I *MtTCPs* showed more widespread and less tissue-/organ-specific expression patterns and had relatively high expression levels in all organs. These results indicated that class I *MtTCPs* might play various regulatory roles at multiple developmental stages.

The class II CYC/TB1 clade *TCPs* are mainly participated in the developmental regulation of axillary meristems, which gives rise to either lateral branches or flowers. This clade included *AtTCP1/12/18* and *MtTCP1A/1B/12*. *MtTCP1A/1B/12* were grouped into a subclade and closely related to *AtTCP1*, although *AtTCP12* was also grouped into this subclade. *AtTCP1*, the ortholog gene of *CYC* in *A. majus*, is involved in the regulation of longitudinal elongation of multiple organs, such as petioles, rosette leaves, and inflorescent stems. *AtTCP1* shows high expression level in inflorescence stem, leaf, and petiole (Koyama et al., 2010b). However, *MtTCP1A, MtTCP1B*, and *MtTCP12* exhibited high transcriptional levels in flower and shoot bud, which is different with that in *AtTCP1*. This finding implies that they may function redundantly in flower and young leaf development in *M. truncatula*. *BRANCHED1* (*BRC1*, also named *AtTCP18*) and *BRANCHED2* (*BRC2*, also named *AtTCP12*), two orthologous genes of *TB1* in maize, were transcribed at high levels in axillary meristems or buds (Aguilar-Martinez et al., 2007; Finlayson, 2007; Niwa et al., 2013). *AtTCP12* shows a lower transcription level and mild mutant phenotype than its homolog *AtTCP18* (Aguilar-Martinez et al., 2007; Finlayson, 2007). The phylogenetically closest gene of *AtTCP18* in *M. truncatula* is *MtTCP18*. *MtTCP18* displayed relatively high expression level in shoot buds. This result suggested that *MtTCP18* is likely to execute similar roles with *AtTCP18* in axillary bud development and branching control.

The class II *TCP* gene *CIN* is expressed downstream of the cell cycle arrest front progression and acts as a repressor of cell proliferation in leaves (Nath et al., 2003). In *Arabidopsis*, the *miR319*-targeted *TCP* genes, *AtTCP2, AtTCP3, AtTCP4, AtTCP10*, and *AtTCP24*, regulate leaf morphogenesis, leaf senescence, and petal growth (Palatnik et al., 2003; Schommer et al., 2008, 2014; Nag et al., 2009; Sarvepalli and Nath, 2011; Li et al., 2012; Bresso et al., 2017; Koyama et al., 2017; Samad et al., 2017). In *M. truncatula*, four closest *MtTCPs* (*MtTCP3, MtTCP4, MtTCP10A*, and *MtTCP10B*) have a putative recognition site of *MtmiR319*. All the *MtmiR319*-targeted *MtTCPs* were highly expressed in leaves. Meanwhile, *MtTCP3* and *MtTCP10A* also exhibited high expression levels in flowers. However, the non-*MtmiR319* target *MtTCP* genes, such as *MtTCP13* and *MtTCP17*, also exhibited similar expression pattern with those of *MtmiR319*-targeted *MtTCPs*, implying the different regulation mechanism controlling their expression.

The root nodule symbiosis is a complex biological process that is controlled by several transcription factors, such as *NSP1*/*2, ERN1*, and *IPN2* (Schauser et al., 1999; Andriankaja et al., 2007; Middleton et al., 2007; Yano et al., 2008; Zhu et al., 2008; Hirsch et al., 2009; Wang et al., 2013; Battaglia et al., 2014; Kang et al., 2014; Singh et al., 2014; Soyano and Hayashi, 2014). Plant hormone cytokinin (CK) plays an essential role in nodulation. CK signaling is required for cell divisions that initiate nodule development (Murray et al., 2007; Tirichine et al., 2007). Previous reports suggest that downregulation of the expression level of *MtLOG1*, a CK riboside 5′-monophosphate phosphoribohydrolase, reduces the nodule number, supporting the positive effect of CK on nodulation (Lohar et al., 2004; Gonzalez-Rizzo et al., 2006; Murray et al., 2007; Tirichine et al., 2007). However, the nodule number also decreases in *35S: MtLOG1* plants, indicating the CK homeostasis is important for nodulation (Mortier et al., 2014). TCPs promote leaf growth and development by modification of responses or sensitivity to CK (Steiner et al., 2012, 2016; Efroni et al., 2013). Overexpression of *MtmiR319A* led to the downregulation of the targeted *MtTCPs*. The transgenic plants exhibited small plants size with reduced nodule number and fresh weight during rhizobial infection, which is similar to the plants with CK deficiency. Therefore, it is possible that *MtmiR319/MtTCPs* module play a significant role in nodule development by regulating the CK pathway.

## Conclusion

In this study, we executed genome-wide analyses and identified *TCP* genes in *M. truncatula*. Those *MtTCP* genes were placed on eight chromosomes with different densities. We characterized *MtTCP* genes expression profiles in different tissues/organs and developmental stages of nodule, suggesting *MtTCP* genes could play vital roles in *M. truncatula* growth and development. In addition, the nuclear-localized signal of four MtTCP-GFP fusion proteins indicated that the MtTCPs were functional transcription factors. Remarkably, overexpression of *MtmiR319A* in *M. truncatula* leads to the decreased nodule number and nodule weight, demonstrating that *MtmiR319/MtTCPs* module might be involved in the regulation of nodule development. Further study is needed to illuminate the molecular mechanism that *MtTCPs* genes involved in leaf and nodule development.

## Author Contributions

HfW and CZ designed the research. HfW and HwW carried out most of the research and analyzed the data. RL and YX performed some nodule analysis experiments. ZL contributed analytical tools. HfW and CZ wrote the paper.

## Conflict of Interest Statement

The authors declare that the research was conducted in the absence of any commercial or financial relationships that could be construed as a potential conflict of interest. The reviewer MB and handling Editor declared their shared affiliation.

## Footnotes

1 http://www.medicagohapmap.org/tools/blastform

2 http://www.Arabidopsis.org/

3 http://rice.plantbiology.msu.edu/index.shtml

4 http://www.medicagogenome.org

5 http://planttfdb.cbi.pku.edu.cn

6 http://www.genome.jp/tools-bin/clustalw

7 http://gsds.cbi.pku.edu.cn

8 http://meme-suite.org/tools/meme

9 http://www.jalview.org

10 http://www.mirbase.org

11 http://plantgrn.noble.org/psRNATarget

12 http://www.Arabidopsis.org

13 http://www.medicagogenome.org

14 http://planttfdb.cbi.pku.edu.cn
