# Supplementary material for: Genome-Wide Identification of TCP Family Transcription Factors in Medicago truncatula Reveals Significant Roles of miR319-Targeted TCPs in Nodule Development
Source: Front Plant Sci. 2018 Jun 11;9:774. doi: 10.3389/fpls.2018.00774 (PMC6004737; doi:10.3389/fpls.2018.00774)
Supplement: Supplementary file 1 [file Image_1.pdf]

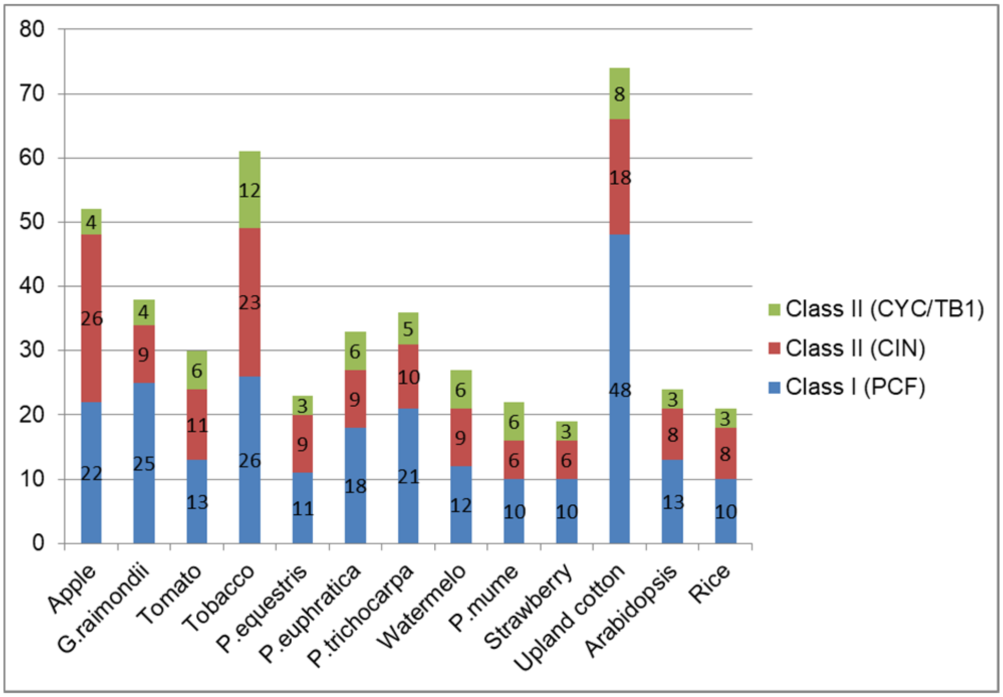

Supplementary Figure 1. Some known TCP family members of different species.

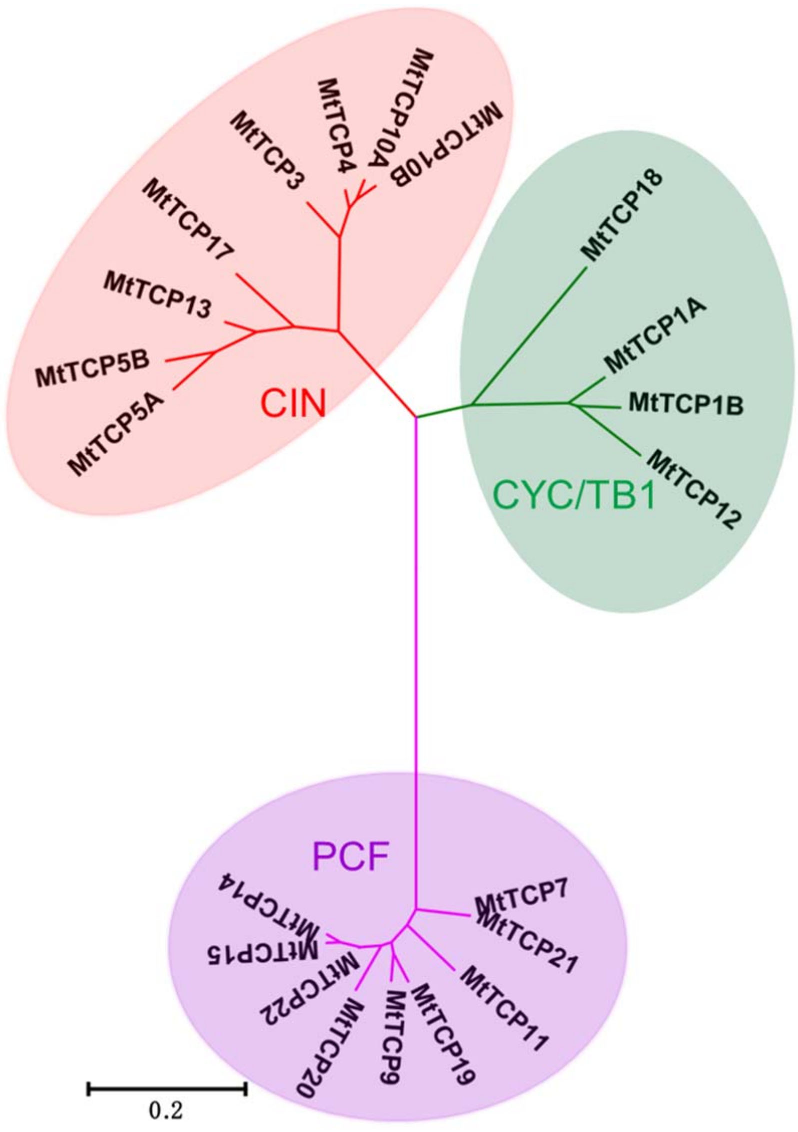

Supplementary Figure 2. Phylogenetic analysis of MtTCP proteins using the TCP domains. Phylogenetic analysis using only the TCP domain amino acid sequences. Multiple sequences were aligned using Clustal W and the phylogenetic trees were constructed using MEGA7.1 by the Neighbor-Joining method with bootstrap replication of 1, 000 times.

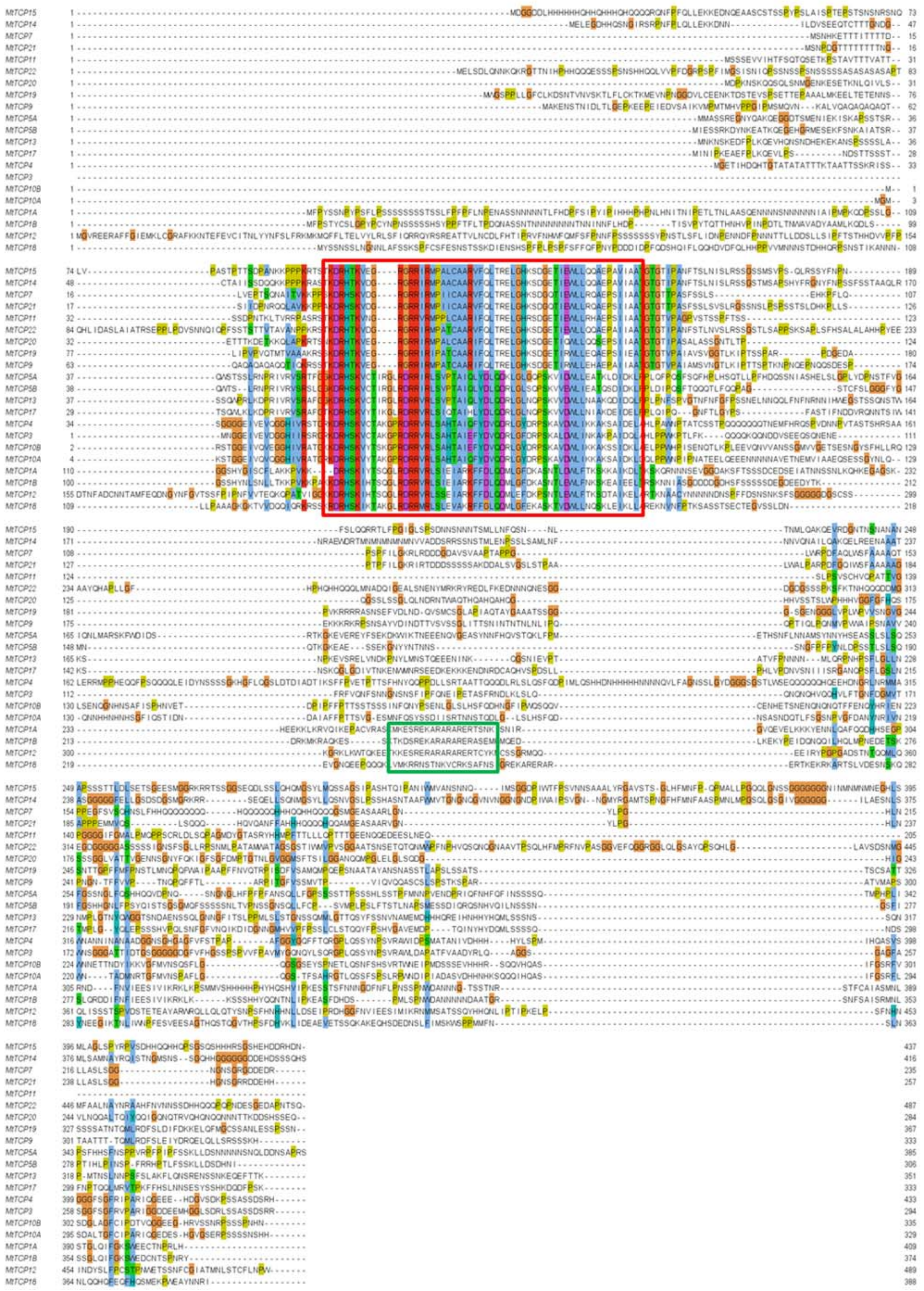

Supplementary Figure 3. Multiple amino acid sequence alignment of MtTCP proteins. Multiple sequence alignment of MtTCP proteins using full-length amino acid sequences**.** Sequences were aligned using Jalview. Red box shows the conserved TCP domain. Green box shows the R domain.

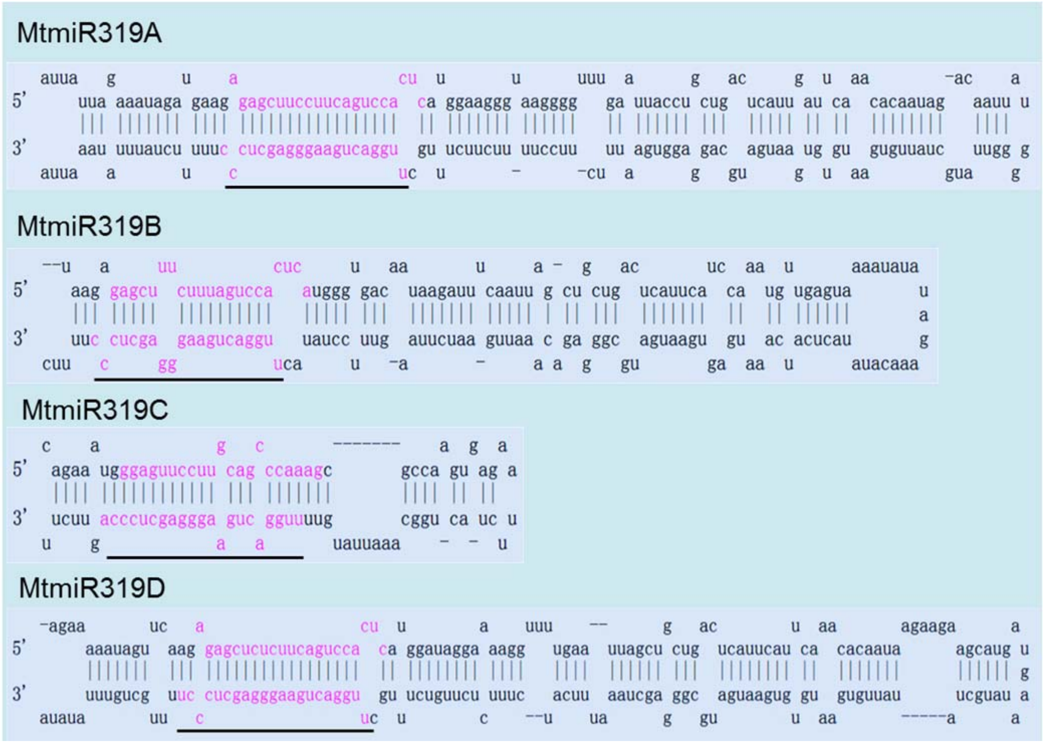

Supplementary Figure 4. Stem-loop structure of the MtmiR319. Website predicted stem-loop structure diagram of MtmiR319. The sequences in pink areas with black lines are the mature sequence of MtmiR319.

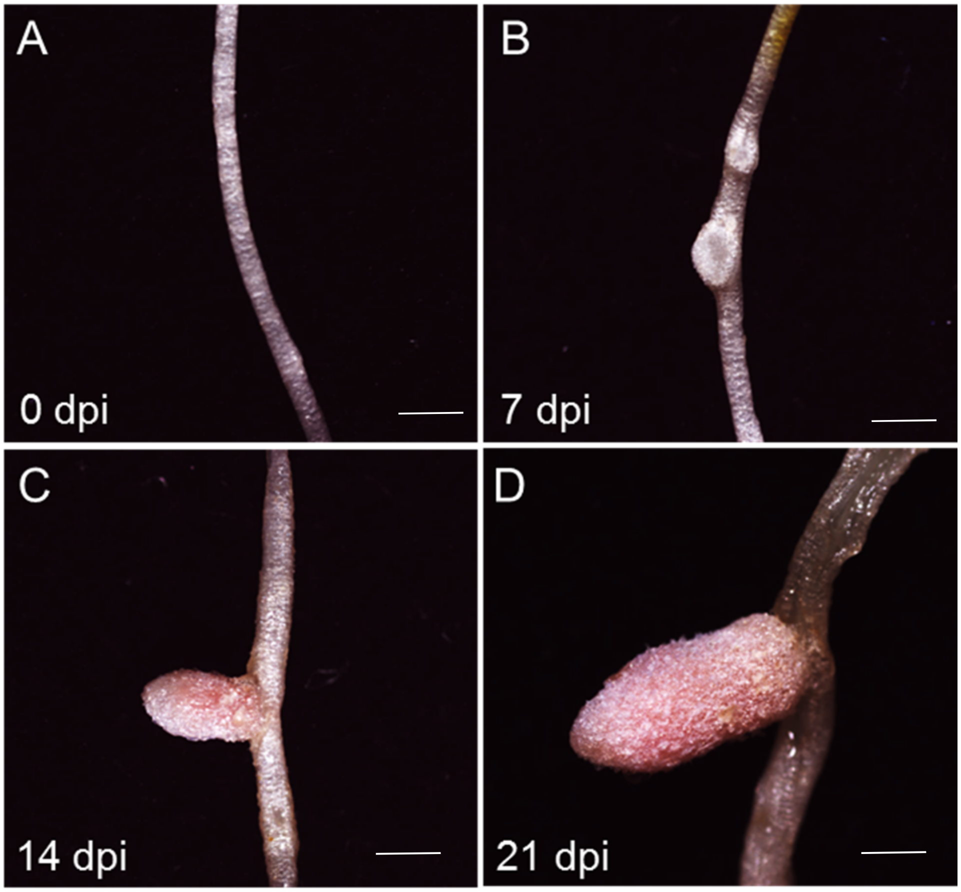

Supplementary Figure 5. Nodules at different developmental stages in wild type. (A-D) The size and shape of nodules at 0, 7, 14, and 21 days post inoculation (dpi) inoculated with *S. meliloti* 1021 strain. Bar = 2 mm.

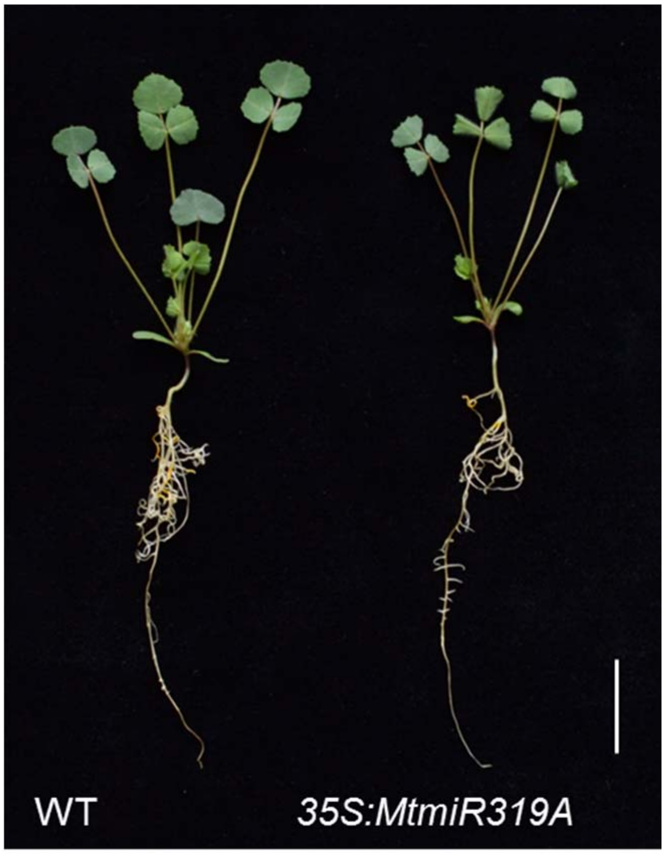

Supplementary Figure 6. Three weeks old plants of wild type and *35S:MtmiR319A* without inoculated with *S. meliloti* 1021 strain. Bar = 1cm.

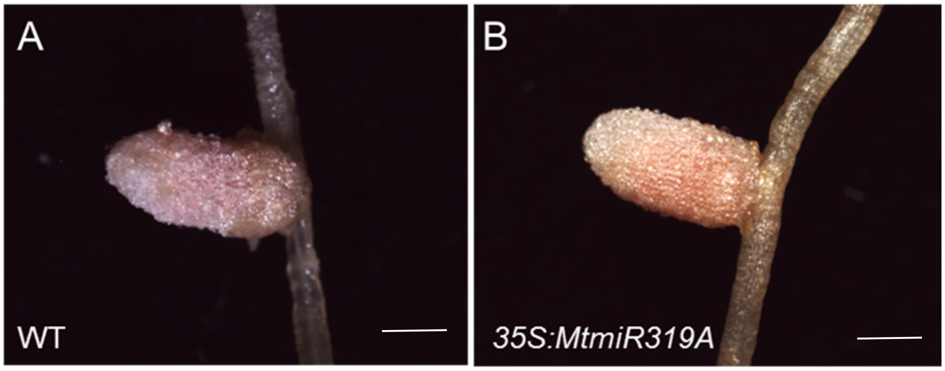

Supplementary Figure 7. Nodules of wild type and *35S:MtmiR319A* plants after three weeks inoculated with *S. meliloti* 1021 strain. Bar = 2 mm.

Supplementary Table 1 Primers used in this study.

MtTCP1A-qRT-F	TTTTGTGCTATAGCCAGCATGAA
MtTCP1A-qRT-R	TCCTCCCATGATTTTCCAAAGA
MtTCP1B-qRT-F	ATAACAATGATGCTGCCACT
MtTCP1B-qRT-R	AAGATTTGAAGCCCTGATG
MtTCP3-qRT-F	TTGTTGCGGCTGATTATCGA
MtTCP3-qRT-R	AAAGCCACCAGAAGCAAATCC
MtTCP4-qRT-F	CAGCGGAGGAGGAGGAGAAA
MtTCP4-qRT-R	CCTGTTGAACGGACAATGTGAC
MtTCP5A-qRT-F	GCAAAGCAAGAAGGAGGTGATAC
MtTCP5A-qRT-R	TGATGTTGACCATTGTCTTGATGTA
MtTCP5B-qRT-F	AACAAACCCTATTTCAGCAACCA
MtTCP5B-qRT-R	TTCATACCATAAAAACCACCTCCAA
MtTCP7-qRT-F	CACAGTAAAGTAGACGGACGAGGAA
MtTCP7-qRT-R	ACGTGCGGCGCAAATTAT
MtTCP9-qRT-F	TGTGCAGCTCGGATCTTTCA
MtTCP9-qRT-R	GTTTCGCCATCGGACTTATGTC
MtTCP10A-qRT-F	TGGTTAATTCGCCTGCGTTT
MtTCP10A-qRT-R	CCCCTATGGGCGGAAAAA
MtTCP10B-qRT-F	CATAGTTCGAGCCACCGGTAA
MtTCP10B-qRT-R	TCGCGAGGACCTTTTGATGT
MtTCP11-qRT-F	AAACCTTCCACCGCCGTAAC
MtTCP11-qRT-R	TGGTGTTGGGATCGGAAGAG
MtTCP12-qRT-F	TGATTACTCCTTATTCCCTTGTTCAA
MtTCP12-qRT-R	TGGCTATTCCACAAAAGTTGGA
MtTCP13-qRT-F	GCTTCATCACAATGGCCAAGAT
MtTCP13-qRT-R	CCTTTCCACCAAAAGCTCTTGA
MtTCP14-qRT-F	CCTGTGCTGCCAGAGTGTTC
MtTCP14-qRT-R	CGTCTCGCCGTCTGATTTATG
MtTCP15-qRT-F	CCGCCGCATCCGAAT
MtTCP15-qRT-R	GCCAAGTTCGCGTGTAAGCT
MtTCP17-qRT-F	CAACCTTCTTTTTTGGGATCGTT
MtTCP17-qRT-R	AGACGACGGCTCCAATTGAT
MtTCP18-qRT-F	GCAAGATCAAAACCGCGAAA
MtTCP18-qRT-R	AAACCTCTTTGCAACTTCAAGTGA
MtTCP19-qRT-F	CACCATGTTTCCTTCAATAGACACCAATGGC
MtTCP19-qRT-R	GTAGCAGAGATAATCGTATAGAG
MtTCP20-qRT-F	CACCATGGATACCGGCCCTTGGAA
MtTCP20-qRT-R	GACCGCCGCCGTCCTAGTT
MtTCP21-qRT-F	CACCATGAATACCGGACCTTGGAGAGATG
MtTCP21-qRT-R	CTTCCGGTCGAACTGGACCGA

MtTCP22-qRT-F	CACCATGGACGGCGGAGACAACG
MtTCP22-qRT-R	ATCTTGTGGATCTTCCTCACCGC
MtTCP3-CDS-F	CACCATGAACGGTGGAGAGATAGTGGAAG
MtTCP3-CDS-R	ACGGCGAGAATCTGAGGACG
MtTCP4-CDS-F	CACCATGGGAGAAACAATACACGACCAGCAC
MtTCP4-CDS-R	ATGGCGAGAGTCGGAGGAAGCAGAG
MtTCP10A-CDS-F	CACCATGAGGAGTACTGGAGGAGAGATAG
MtTCP10A-CDS-R	GTTGTGATTAGGAGAAGAAGAA
MtTCP10B-CDS-F	CACCATGGGAATGAAGAGCACAGGAGG
MtTCP10B-CDS-R	GTGATGAGAATTAGAAGAAGAGG
MtmiR319A-F	CACCATTTAGGGTTAGGGTTCTTTGTTTG
MtmiR319A-R	TTGGTCTAACCTCGCTACATCAC
MtUBI-qRT-F	CTGACAGCCCACTGAATTGTGA
MtUBI-qRT-R	TTTTGGCATTGCTGCAAGC
